# DNA methylation signatures of Prostate Cancer in peripheral T-cells

**DOI:** 10.1186/s12885-020-07078-8

**Published:** 2020-06-23

**Authors:** Ali Mehdi, David Cheishvili, Ani Arakelian, Tarek A. Bismar, Moshe Szyf, Shafaat A. Rabbani

**Affiliations:** 1 Department of Medicine, McGill University, Montreal, Quebec Canada; 2 Department of Human Genetics, McGill University, Montreal, Quebec Canada; 3 HKG Epitherapeutics, Hong Kong, China; 4 Department of Oncology, McGill University, Montreal, Quebec Canada; 5 Departments of Pathology & Laboratory Medicine, Oncology, Biochemistry & Molecular Biology, Cumming School of Medicine, University of Calgary, Calgary, Alberta Canada; 6 Department of Pharmacology, McGill University, Montreal, Quebec Canada; 7 McGill University Health Centre, 1001 Décarie Blvd. (Glen site), Room EM1.3232, Montréal, QC H4A3J1 Canada

**Keywords:** Prostate cancer, DNA methylation, T cells, Blood, Diagnosis, Immune

## Abstract

**Background:**

Prostate Cancer (PCa) is the second most common cancer in men where advancements have been made for early detection using imaging techniques, however these are limited by lesion size. Immune surveillance has emerged as an effective approach for early detection and to monitor disease progression. In recent studies, we have shown that host peripheral blood immune cells undergo changes in DNA methylation in liver and breast cancer.

**Methods:**

In the current study, we examined the DNA methylation status of peripheral blood T cells of men with positive biopsy for PCa versus men with negative biopsy having benign prostate tissue, defined as controls. T cells DNA was isolated and subjected to Illumina Infinium methylation EPIC array and validated using Illumina amplicon sequencing and pyrosequencing platforms.

**Results:**

Differential methylation of 449 CG sites between control and PCa T cell DNA showed a correlation with Gleason score (*p < 0.05*). Two hundred twenty-three differentially methylated CGs between control and PCa (∆ß +/− 10%, *p < 0.05*), were enriched in pathways involved in immune surveillance system. Three CGs which were found differentially methylated following DMP (Differentially methylated probes) analysis of ChAMP remained significant after BH (Benjamini-Hochberg) correction, of which, 2 CGs were validated. Predictive ability of combination of these 3 CGs (polygenic methylation score, PMS) to detect PCa had high sensitivity, specificity and overall accuracy. PMS also showed strong positive correlation with Gleason score and tumor volume of PCa patients.

**Conclusions:**

Results from the current study provide for the first-time a potential role of DNA methylation changes in peripheral T cells in PCa. This non-invasive methodology may allow for early intervention and stratification of patients into different prognostic groups to reduce PCa associated morbidity from repeat invasive prostate biopsies and design therapeutic strategy to reduce PCa associated mortality.

## Background

Prostate Cancer (PCa) is the second most common cancer in men and has been estimated to affect more than a million men worldwide in 2018 [1–4]. PCa is graded using the Gleason score (ranging from 2 to 10) that examines the differentiation of cancer cells according to their histological pattern [1–5]. In its early stage, tumor cells are androgen sensitive however, in advanced stages they are androgen insensitive, metastasize to distant organs and have limited therapeutic strategies available [3, 5]. Therefore, there is an urgent need to identify sensitive biomarkers which can detect initial and recurrent PCa early to allow for effective intervention. Diagnosis for PCa includes digital rectal examination and determination of the levels of prostate specific antigen however, each of these tests have limitations [6, 7]. On the other hand, prostate biopsies are invasive, can provide false-negative results and do not examine the whole prostate [5, 6, 8, 9].

DNA methylation has emerged as a major area of investigation for the identification of cancer specific biomarkers at an early time point which can be utilized as a diagnostic or prognostic biomarker in numerous cancers including PCa [10–18]. DNA methylation alterations are highly common and are particularly able to provide a high degree of sensitivity using plasma and urine of PCa patients [12, 19]. However, these studies were limited in scope due to their focus on a single or few gene loci. Collectively a multi-faceted approach is required to accurately predict and detect PCa for early intervention and develop effective therapeutic strategies.

Host immuno-surveillance plays an important role in the recognition and elimination of transformed and tumor cells [20–22]. T-cells are the most prominent component of this system that control tumor growth [20–22] and hence have the potential of being effective cancer biomarkers [23–25]. In previous studies, we have identified a DNA methylation signature in peripheral blood mononuclear cells and T cells in hepatocellular carcinoma (HCC) which distinguished HCC from chronic hepatitis B and C versus healthy controls [25]. Similarly, DNA methylation signature in T cells of breast cancer patients showed strong correlation with breast cancer progression compared to healthy controls [24].

In the current study, we have carried out genomic DNA methylation assay using peripheral blood T cells from negative-biopsy men having benign prostate tissue, defined as controls, and positive biopsy PCa patients. T cells DNA isolated from control and PCa subjects was analyzed by Illumina Infinium methylation EPIC array platform and validated using amplicon- and pyro- sequencing. We have identified a DNA methylation signature that not only distinguishes healthy controls from PCa patients but also different stages of PCa according to Gleason score.

## Methods

### Study populations

The study design was approved by the ethics committee of Alberta PCa Research Initiative (APCaRI). Buffy coats for positive-biopsy PCa patients and healthy negative-biopsy, having benign prostate tissue defined as controls, men were obtained through APCaRI. All patients were enrolled at the time of diagnosis before initiation of any treatment and had strict exclusion criterion of any autoimmune disease, asthma or infection which could alter T cells characteristics. The study had 12 controls and 20 PCa samples; 9 of which were low Gleason PCa (LGPCa) having a Gleason score ≤ 7 and; 11 of which were high Gleason PCa (HGPCa) having a Gleason score > 7 (Cohort 1) (Supplementary Figure 1A and Supplementary Table 1). We used an older cohort (Cohort 2) which had 11 controls and 13 LGPCa samples for some analysis (described below) (Supplementary Table 1). The controls (mean age of 58.0 years) and the PCa patients (mean age of 62.4 years) had no significant difference between mean age (*p > 0.05*). The percentage tumor volume (%) was calculated as the total volume of positive cores divided by all cores tissue and is reflective of the potential cancer volume in the prostate gland.

### T-cell isolation, Methylome assay and pyrosequencing

Firstly, CD3+ T cells were isolated using anti-CD3 immuno-magnetic beads (Dynabeads®, Invitrogen) from the buffy coats of PCa and healthy controls according to manufacturer’s protocol. Genomic DNA was extracted from the T cells using AllPrep DNA/RNA mini kit (Qiagen, Canada) according to manufacture’s protocol as previously described [24, 25].

Methylome assay was performed on the genomic DNA (gDNA) extracted from T cells using Infinium® MethylationEPIC BeadChip assay from Illumina (Illumina Inc., CA, USA). Following sodium bisulfite conversion of gDNA Infinium® MethylationEPIC BeadChip assay was carried out as previously described [24, 25].

Pyrosequencing was performed according to PyroMark Q24 (QIAGEN) following manufacturer’s protocol except a few modifications according to Tost et al [24–26] All data were expressed as mean ± standard error of the mean (SEM). Primers used for the analysis are listed in Supplementary Table 2.

### Amplicon sequencing (using Illumina MiSeq)

The bisulfite converted DNA was quantified by a Nanodrop Spectrophotometer (Thermo Fisher Scientific, MA, USA) followed by two rounds of polymerase chain reaction (PCR) targeting the cg14713996 and cg05133736 regions using Bio-Rad C1000 Touch Thermal Cycler (Bio-Rad Laboratories, CA, USA) for multiplex sequencing. The pooled library was then purified twice using AMPure XP Beads (Beckman Coulter Life Sciences, CA, USA) and quantified by RealTime PCR using NEBNext® Library Quant Kit for Illumina (New England Biolabs, MA, USA) and sequencing was performed on the Illumina platform using MiSeq Reagent Nano Kit V2 (Illumina, CA, USA).

### Data and statistical analysis

Data analysis was performed in R software using the ChAMP pipeline from Bioconductor at default setting and quality controlled using BMIQ and SVD while batch corrected with ComBat [27, 28]. Indeed, significant (*P < 0.05*) variation arising from the Slide variable were completely removed after running the ComBat function (Supplementary Figure 2). We carried out further QC as previously described by us and others [24, 25]. Subsequently, Differentially Methylated Probes (DMPs) between PCa and negative-biopsy controls was run in ChAMP with Benjamini-Hochberg (BH) and False Discovery Rate (FDR) as < 0.05. We performed Pearson correlation between the normalized DNA methylation beta values and Gleason score using the Pearson correlation function in R and corrected for multiple testing using BH method (FDR < 0.05). Next, we used an older small cohort (cohort 2) for which we had 11 control and 13 LGPCa T cell DNA samples (Supplementary Table 1). For this cohort, we had run Illumina Infinium Human Methylation 450 K BeadChip in the past but there were no significantly differentially methylated CGs between the groups after BH correction (FDR < 0.05). However, several CG methylation levels were significantly correlated with the Gleason score. Hence, we combined the CGs in 450 K and 850 K cohorts that correlated with the Gleason score and plotted a Heatmap and Boxplot of these CGs.

We shortlisted differentially methylated CGs which had delta-beta value of +/− 10% in between the groups and plotted a Heat-map. Then the genes associated with these CGs were subjected to a pathway analysis using ConsensusPath DB (with *p < 0.01* and *q < 0.05*) [29]. The flow chart of experimental approaches are outlined in Supplementary Figure 1B. Heatmaps were generated using GENE-E of broad institute [30].The statistical analyses were performed using the computing environment R version 3.4.4 and GraphPad Prism 6.0 (GraphPad Software Inc., California, US).

## Results

### Differentially methylated genes in T-cells of PCa patients are enriched in immune-related pathways

**Fig. 1:**
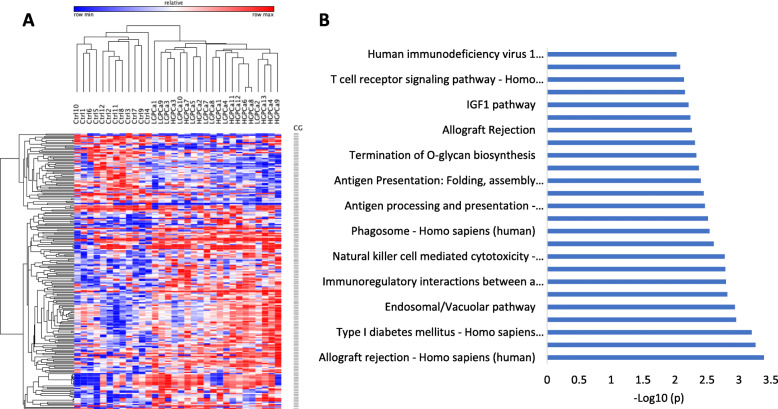
Differentially methylated CGs and respective gene-related pathways deregulated in PCa compared to the controls. A total of 223 statistically significant CGs which had 10% methylation difference in T cell DNA between Ctrl and PCa (*p < 0.05*). **a** Heatmap of the 223 CGs shown for each sample. The scale bar shows methylation levels where highest is 100% and lowest is 0% of that CpG; Blue; hypomethylation; Red; hypermethylation. **b** Differentially methylated genes nearby 105 CGs showed various pathways involved in immune system and immuno-surveillance system. These pathways were obtained by conducting an overrepresentation analysis of the genes nearby 105 CGs utilizing ConsensusPath DB. The pathways have been plotted against -log10 of the *p* value

We delineated 223 differentially methylated CGs between the two groups of healthy individuals and PCa patients, using the Bioconductor package Limma, implemented in ChAMP [31, 32] with a delta beta threshold of +/− 10% (Supplementary File S1) and plotted a heatmap (Fig. 1a). The differentially methylated genes were subjected to pathway analysis. Notably, genes associated with differentially methylated CG sites were enriched in the pathways involved in immuno-editing and immune-surveillance systems (Fig. 1b).

### Correlation between DNA methylation levels and Gleason score in PCa

**Fig. 2:**
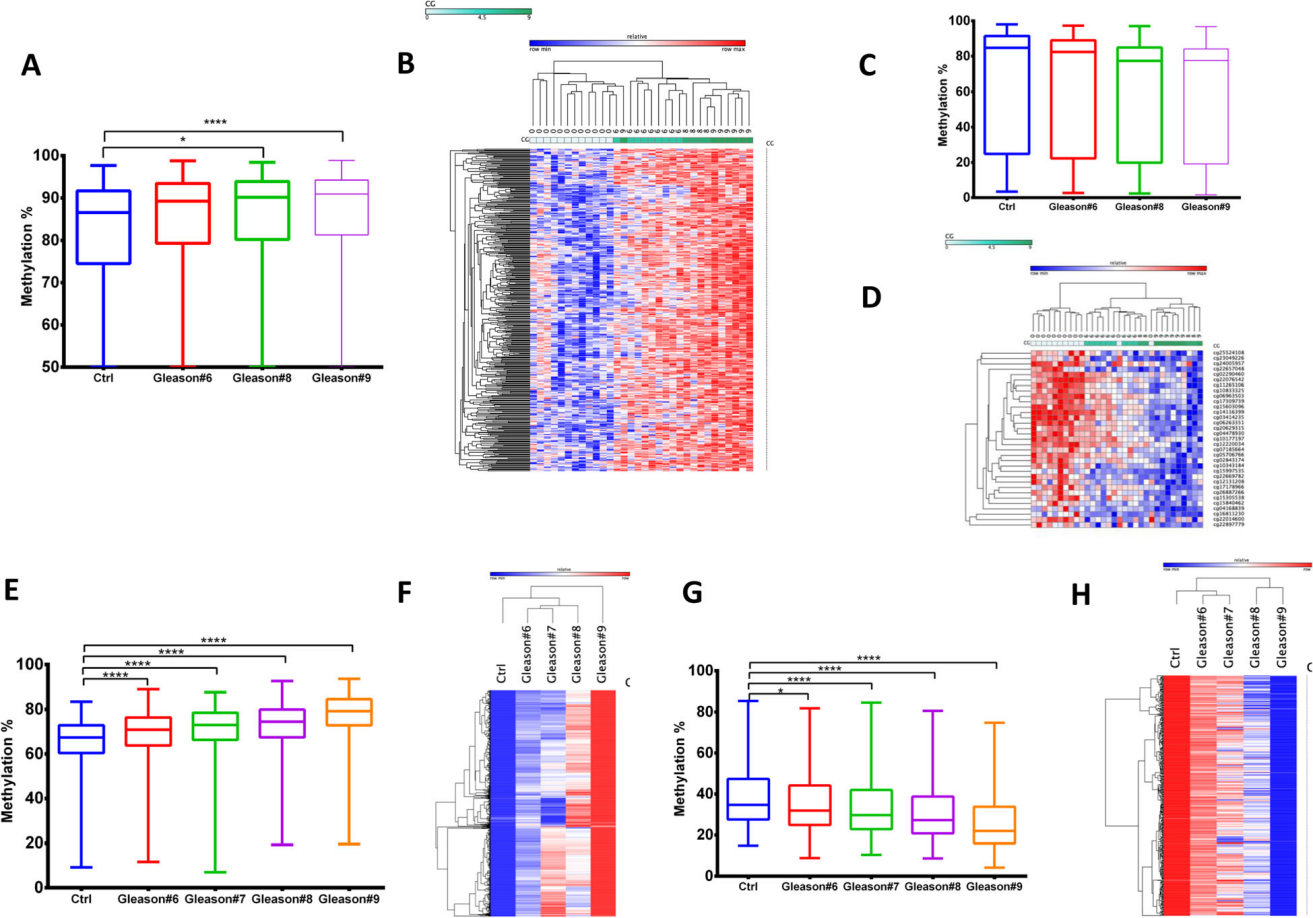
Pearson correlation between CG methylation levels (delta beta> 0.1) and Gleason score. **a**-**d** A total of 449 CGs’ T cell DNA methylation levels showed to be significantly (Q < 0.05 after FDR correction) correlated with Gleason score (0 for healthy individuals and 6, 8 and 9 for Gleason score 6, 8, 9). Boxplots of mean percentage methylation of the 416 and 33 CGs increasing or decreasing with respect to Gleason score, respectively. **b** and **d** Heatmaps of the methylation levels of 416 and 33 CGs that correlate with Gleason score either positively or negatively, respectively. **e**-**h** A total of 1181 CGs whose methylation levels significantly (Q < 0.05) correlated with Gleason score when the two cohorts, Cohort 1 and 2, were combined (Combined cohort). (E and F): Boxplots of mean percentage methylation of the 749 and 432 CGs increasing or decreasing with respect to Gleason score, respectively (G and H). Heatmaps of methylation levels of 749 and 432 CGs that correlate with Gleason score either positively or negatively, respectively

To delineate a set of differentially methylated CGs that are correlated with Gleason score progression, we performed a Pearson correlation analysis (Hmisc R) (0 for healthy individuals and 6, 8 and 9 for Gleason score 6, 8, 9). We corrected for multiple testing using the FDR (Q of < 0.05) Benjamini-Hochberg method. This analysis revealed a signature of DNA methylation comprised of 449 CGs which correlates with Gleason scores in T cells from PCa patients (Fig. 2a-d). Four hundred sixteen CGs, out of 449 CGs, were hypermethylated (Fig. 2a and b) and 33 were hypomethylated (Fig. 2c and d). Boxplot of the methylation values of the differentially methylated sites included in the signature demonstrates the magnitude and progressive change in average DNA methylation as the Gleason score progresses from normal individuals to PCa patients with the highest differences observed at the highest Gleason score of 9 (Fig. 2a and c). Heatmap and hierarchical clustering analysis of 416 hypermethylated and 33 hypomethylated sites accurately grouped normal and PCa patients with Gleason score 6, 8 and 9 with exception of one PCa patient in Fig. 2b and two normal in Fig. 2d.

We next performed a Pearson analysis between methylation levels and Gleason scores of 354,692 CG sites that were also included in a 450 K Illumina bead array analysis of a different cohort (cohort 2) which we performed earlier (hereafter Combined cohort) (Supplementary Table 1). Eleven thousand seven hundred twenty-two CG sites showed a statistically significant correlation after FDR correction (Supplementary File S2); 8478 were hypermethylated and 3244 were hypomethylated. We shortlisted 1181 (Supplementary File S3) progressively methylated CGs that had a 10% difference in methylation level between average DNA methylation of PCa patients with Gleason score 9 and in normal individuals. Seven hundred forty-nine out of 1181 were hypermethylated and 432 hypomethylated (Fig. 2e-h). As demonstrated by the heatmap in Fig. 2f and h, the methylation profile correlates with increasing Gleason score from 6, 7, 8 to 9. We next overlapped 184 out of 449 differentially methylated CGs from EPIC (850 K) cohort with 11,722 differentially methylated in combined cohort (265 CGs were excluded as being specific to EPIC array CG list). This analysis resulted in 89 CGs that were significantly correlated with Gleason score in both (hypergeometric, *p = 1.56e-90*).

A genome-wide distribution on IGV browser [33] of 1181 differentially methylated CGs whose methylation changed progressively with Gleason score (*p < 0.05*) is shown in Supplementary Figure 3. Most sites were hypermethylated, as Gleason score progress from 6 to 9, while a small fraction was hypomethylated. These data are consistent with our previous data in breast and liver cancer and support the hypothesis that epigenome-wide methylation changes occur in T cells genome as the cancer progress [24, 25]. Interestingly, there was significant overlap (*p = 8.93e-42*) between the list of progressively changed methylation of CG sites as cancer progresses in prostate and liver, but not between prostate and breast or breast and liver (Supplementary Figure 4).

### Differentially methylated regions (DMRs)

We analyzed differentially methylated regions (DMRs) utilizing the DMR function in ChAMP pipeline. We found 10 DMRs between the PCa patients and controls (Supplementary Table 3 and Supplementary Figure 5). We visualized these DMRs in IGV using the reference genome Human Hg19 to identify genes overlapping with or nearby these regions (Supplementary Table 3). The DMRs were present near genes at different sites including transcriptions start sites (TSS), intergenic and intragenic regions. Pathway analysis on these genes showed only apoptosis pathway significantly different from control samples (data not shown). Although DMR function provides a good overview of DMRs around the genome, however, we were interested in CGs that could distinguish between healthy and PCa patients specifically. Thus, we moved to differentially methylated probes (DMPs) analysis in ChAMP pipeline.

### Differentially methylated probes (DMPs) between PCa and normal progressively change with Gleason score

**Fig. 3:**
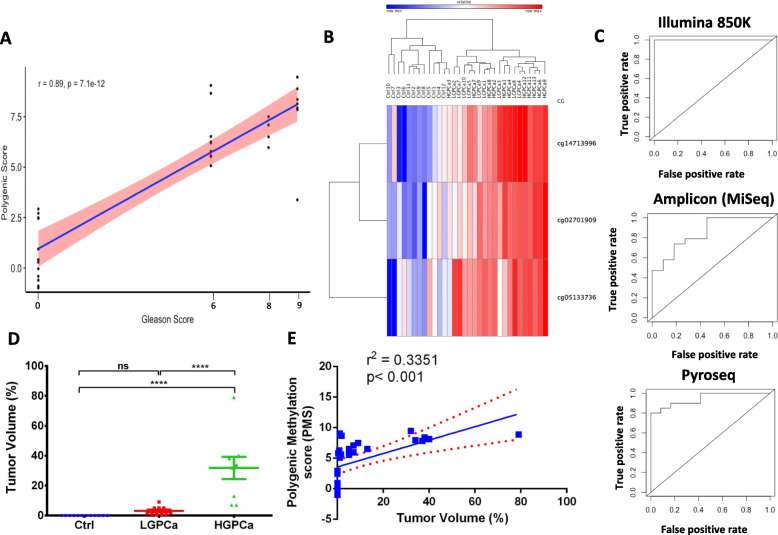
Polygenic methylation score (PMS) for detection of PCa. **a** Pearson correlation analysis of a weighted PMS of three target CGs (DMPs) in T cell DNA and Gleason score. DMPs were identified using ChAMP package in R. These three DMPs were combined to calculate a PMS for each sample which was plotted against Gleason score using a multivariate linear regression model as described in the Results section. **b** Heatmap of the T cell DNA methylation levels of the 3 DMPs for all negative-biopsy controls and positive-biopsy PCa patients (High methylation Red; Low methylation, Blue). **c** ROC curve showing the sensitivity and specificity of PMS score. **d** Mean percentage tumor volume (%) of control (Ctrl), LGPCa (Low Gleason PCa) and HGPCa (High Gleason PCa) samples predicted from core prostate biopsies as described in Methods. Ctrl (Control, Blue); LGPCa (Red); and HGPCa (Green). **e** Linear regression analysis of the PMS from T cell DNA with percentage tumor volume. The red dotted lines show the 95% confidence band of the line of best fit. The negative-biopsy control samples were assumed to have a tumor volume of 0% and a Gleason score of 0

We next performed differentially methylation analysis between healthy individuals and PCa patients with both, high and low Gleason score (HGPCa and LGPCa) using stringent criteria of keeping BH correction at FDR < 0.05. This analysis resulted in three significant CGs (cg14713996, cg02701909, cg05133736) whose methylation levels discriminated between healthy controls and PCa patients (Table 1 and Fig. 3).

**Table 1:** Differentially methylated probes (DMPs) between negative-biopsy controls and positive-biopsy PCa patients identified by Illumina EPIC methylation assay

CpG	Chromosome Location	Gene nearby	Delta Beta /LogFC	FDR	Site of differential methylation	CGI
**cg14713996**	X		0.12	0.01	IGR	opensea
**cg02701909**	1	CTRC	0.03	0.03	1stExon	opensea
**cg05133736**	10		0.08	0.04	IGR	opensea
**cg06915331**	11	PDE2A	0.03	0.12	Body	shore
**cg01235591**	13	ATP11A	0.03	0.12	3’UTR	shore

We then determined whether a polygenic methylation score of these 3 sites could serve as an accurate predictor of PCa. We calculated the regression coefficient of each CG and intercept using a multivariate linear regression model that included three methylation positions: polygenic methylation score (PMS) = ([β1*27.184 + β2*28.842 + β3*24.57)-58.774]; where β1 = methylation level of cg14713996, β2 = methylation level of cg02701909 and β3 = methylation level of cg05133736). This polyvariable linear regression equation, composed of weighted methylation level of three CG sites, was highly significant for the prediction of Gleason score in PCa patients (*r* = 0.89, *p = 7.1e*^*− 12*^) as demonstrated in Fig. 3a and b.

We generated receiver operating characteristic (ROC) curves and measured sensitivity, specificity and the area under the ROC curve (AUC) for each CG (cg14713996, cg02701909, cg05133736) and for PMS. Figure 3c shows ROC curves plotting the clinical performance of polygenic methylation markers for the detection of PCa. The combination of the 3 CGs (PMS) had higher sensitivity, specificity and overall accuracy particularly using Illumina EPIC (Table 2 and Fig. 3c).

**Table 2:** Sensitivity, specificity, accuracy and the area under the ROC curve (AUC) for each CG and for PMS, tested for Illumina EPIC (850 K), amplicon sequencing and pyrosequencing platforms

		Sensitivity	Specificity	Accuracy	AUC
**Illumina 850 K**	cg14713996	0.95	0.92	0.94	0.98
	cg02701909	0.95	0.92	0.94	0.98
	cg05133736	0.9	0.92	0.9	0.97
	PMS	1	1	1	1
**Pyrosequencing**	cg14713996	0.95	0.83	0.9	0.95
	cg02701909	na	na	na	na
	cg05133736	0.8	0.83	0.81	0.8
	PMS	0.85	0.92	0.87	0.95
**Amplicon sequencing**	cg14713996	0.79	0.82	0.8	0.83
	cg02701909	na	na	na	na
	cg05133736	0.85	0.66	0.78	0.73
	PMS	0.74	0.82	0.83	0.85

Percentage tumor volume, which is reflective of the potential cancer volume in the prostate gland of PCa patients, also showed a strong significant positive correlation with Gleason score (Supplementary Figure 6A; *r*^*2*^ = 0.4032; *p < 0.0001*). However, the data points best fit was a non-linear exponential curve (Supplementary Figure 6B; *r*^*2*^ = 0.8344). Indeed, when stratified into healthy controls (Ctrl), LGPCa and HGPCa, the HGPCa showed significantly higher mean tumor volumes (%) than both the other groups (Fig. 3d). Moreover, PMS also showed strong significant positive correlation with tumor volume (Fig. 3e; *r*^*2*^ = 0.3351; *p < 0.001*) indicating that PMS was able to predict progressiveness of the tumor. Hence, a higher PMS in peripheral T cells’ DNA would potentially indicate a higher tumor volume in the prostate of that patient and vice versa.

### Validation of differentially methylated cg14713996 and cg05133736

**Fig. 4:**
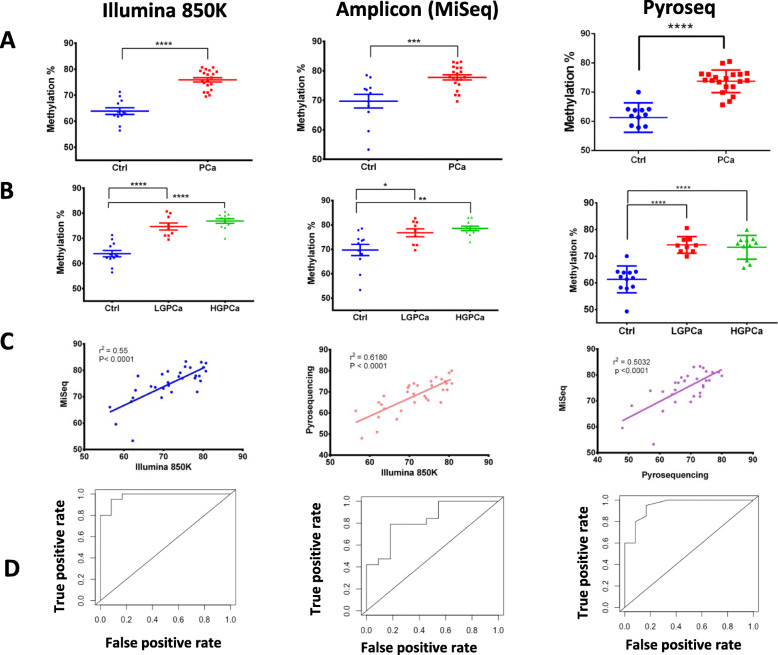
Identification and validation of cg14713996 as a DNA methylation marker using Illumina EPIC (850 K), amplicon sequencing (using MiSeq system) and pyrosequencing. **a**-**b** Percentage methylation of T cell DNA of each sample at cg14713996 plotted as; **a** Ctrl (Control, Blue) and PCa (PCa, Red); **b** Ctrl (Control, Blue), LGPCa (Low Gleason PCa, Red) and HGPCa (High Gleason PCa, Green). Statistical significance was obtained in GraphPad Prism and are represented by asterisks (*P < 0.05; **P < 0.01, ***P < 0.001, ****P < 0.0001). **c** Correlation graphs between Illumina EPIC (850 K), amplicon sequencing and pyrosequencing. **d** ROC curve showing the sensitivity and specificity of cg14713996 for detection of PCa

**Fig. 5:**
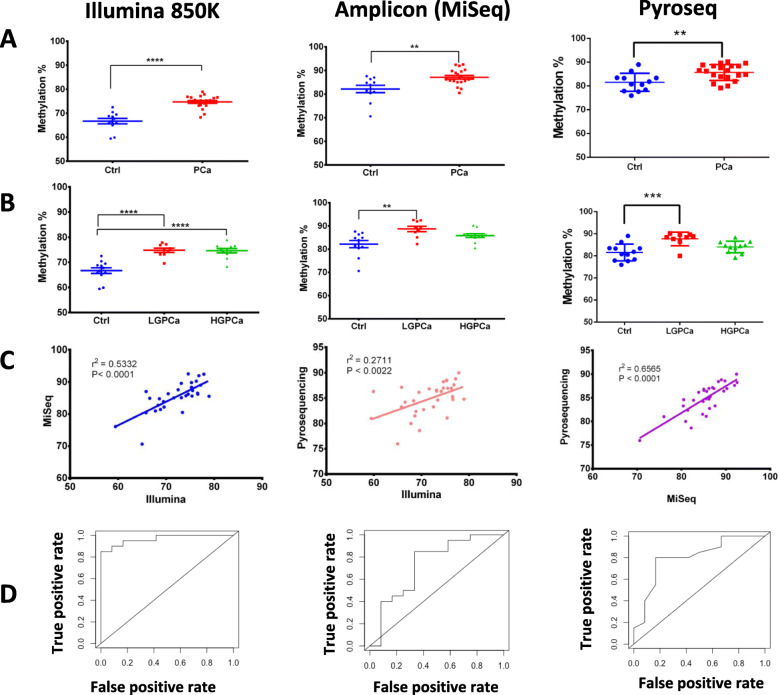
Identification and validation of cg05133736 as a DNA methylation marker using Illumina EPIC (850 K), amplicon sequencing (using MiSeq system) and pyrosequencing systems. **a**-**b** Percentage methylation of T cell DNA of each sample at cg05133736 plotted as; **a** Ctrl (Control, Blue) and PCa (PCa, Red); **b** Ctrl (Control, Blue), LGPCa (Low Gleason PCa, Red) and HGPCa (High Gleason PCa, Green). Statistical significance was obtained in GraphPad Prism and are represented by asterisks (*P < 0.05; **P < 0.01, ***P < 0.001, ****P < 0.0001). **c** Correlation graphs between Illumina EPIC (850 K), amplicon sequencing and pyrosequencing. **d** ROC curve showing the sensitivity and specificity of cg05133736 for detection of PCa

We next validated cg14713996 and cg05133736 using pyrosequencing and amplicon sequencing and due to insufficient amount of T cells DNA we couldn’t validate cg02701909 for amplicon sequencing and some samples for pyrosequencing (Fig. 4, Fig. 5 and Supplementary Figure 7). The validation set included 20 PCa patients and 12 healthy controls.

cg14713996 showed a 12% (± 1.45%) difference in methylation between the controls and PCa in the Illumina EPIC (850 K) assay (Fig. 4a; Table 2). The methylation level of this site progressively changes between controls, LGPCa and HGPCa (Fig. 4b). We examined correlation between Illumina EPIC array, amplicon sequencing and pyrosequencing assays (*r*^*2*^ = 0.55 and *r*^*2*^ = 0.618, respectively, *P < 0.0001*)*,* as well as amplicon sequencing and pyrosequencing (*r*^*2*^ = 0.5032, *P < 0.0001*) (Fig. 4c).

cg05133736 showed an 8% (± 1.15%) difference in DNA methylation between the controls and PCa in the Illumina EPIC (850 K) assay (Fig. 5a; Table 2). Validations and correlations were performed for cg05133736 as well (Fig. 5a-c). Lastly, both cg14713996 and cg05133736 had high sensitivity, specificity and overall accuracy particularly using Illumina EPIC (Table 2, and Figs. 4d and 5d, respectively).

## Discussion

DNA methylation alterations in leukocytes have been reported by us and others in many cancers including head and neck squamous cell carcinoma, ovarian [34, 35], colorectal [36], breast [24], and HCC [25, 37]. We focused on T cells which are essential for controlling tumorigenesis and epigenetic changes like DNA methylation which are likely to occur first in T cell as they are activated even before malignant transformation. Detection of these approaches which are highly sensitive, specific and non-invasive will allow stratification of men for the risk of developing or having PCa [24, 25]. In the current study, identified genes and CGs were enriched in signaling pathways implicated in immune-editing and -surveillance which allowed differentiation of control and PCa subjects, and also PCa subjects with various Gleason scores. Consistent with immune-editing theory, these results provide additional support that the DNA methylation signature can be utilized in assessing risk of early stages of PCa.

Previous studies have shown differential methylation of individual gene promoters in PCa. A study investigating clinical significance of gene promoter DNA methylation in blood DNA found six genes promoter methylation of PCa patients to be significantly different from controls [38]. However, we did not see such differences which could be due to several reasons; firstly, T cells DNA was used in our study while blood DNA was used in previous study [38]; secondly, previous study was carried out in African American men while our study had mostly Caucasian men [38]. Also, using whole blood DNA, no association between the DNA methylation on all CG sites and risk of PCa or aggressive disease was observed [39]. Similarly, in a small cohort, we also did not find any CG site with differential methylation between controls and PCa patients T cell DNA (data not shown) which could be due to small sample size and use of Illumina 450 K which does not include FANTOM5 and ENCODE enhancer regions and many other sites that were added in EPIC (850 K) array [40]. Our previous studies in breast and liver cancer validated the use of T cells to distinguish between control and cancer and also differentiated between different disease stages [24, 25]. Similarly, our study is not only consistent in distinguishing between negative and positive biopsy for PCa but also according to Gleason score.

Despite these convincing results we are aware of the limitation of our study due to the small sample size. However, these results are robust in differentiating normal and PCa subjects, identifying several CGs which were validated using amplicon sequencing and pyrosequencing [26, 27, 41–43]. In addition, our previous study involving T cells from breast cancer had smaller sample size of 19 samples, but was able to distinguish between healthy controls and cancer patients [24]. Our DNA methylation signature gets stronger between controls and PCa patients across various Gleason scores and the highest difference is seen between Gleason score of 9 and control subjects. Importantly, the PMS was able to not only predict PCa patients from healthy controls, but also Gleason score indicating progressive stages of PCa with strong predictive power of PMS with high sensitivity, specificity and accuracy (Fig. 3c and Table 2). Indeed, the PMS in the T cells’ DNA showed strong correlation with tumor volume of PCa patients that is strongly associated with Gleason score as showed herein and previously (Fig. 3 and Supplementary Figure 6). While all subjects had no history of immune disorders or infection, due to the samples size we were unable to take into account additional confounding states including smoking and alcohol intake which can affect the state of DNA methylation [44–48].

Results of this study are the first to identify the DNA methylation signature in immune cells of PCa subjects using non-invasive approach for early detection and stratification of PCa. It is imperative to carry out follow up studies using a larger cohort of subjects which will also consider history of smoking, alcohol intake and stratify the subjects based on their racial background. Future studies will also monitor differences among different T cells, PCa sub-types, stages and Gleason score. It is anticipated that the T cell DNA methylation signature will be identifiable in whole blood for large scale screens, active surveillance, early intervention with targeted therapies including the use of epigenetic based therapeutic agents.

## Conclusions

The current study provides for the first-time a potential role of DNA methylation changes in peripheral T cells in PCa. Using this non-invasive approach, changes in the DNA methylation status of T cells can predict men at risk of developing PCa and disease progression. Furthermore, the non-invasive methodology may allow for early intervention and stratification of patients into different prognostic groups to reduce PCa associated morbidity from repeat invasive prostate biopsies and design therapeutic strategy to reduce PCa associated mortality.

## Supplementary information

**Additional file 1: Supplementary Figure 1.** (A) The number of T cell DNA samples used in the cohort 1. Ctrl, Negative-biopsy healthy control; LGPCa, Positive-biopsy low Gleason PCa; HGPCa, Positive biopsy high Gleason PCa. (B) Outline of DNA methylation signature determination in DNA of peripheral T cells in buffy coat of PCa patients and healthy controls using Illumina Infinium EPIC methylation array followed by validation with Illumina MiSeq and Pyrosequencing platforms. **Supplementary Figure 2.** Quality control (QC) of the data from Illumina Infinium EPIC methylation array using QC in ChAMP pipeline. (A) Density plot of raw data showing the beta distributions for each healthy control and PCa sample. None of the samples deviate from each other. (B) Singular value decomposition analysis (SVD) plot shows the significant components of variation in T cell DNA methylation. SVD analysis before (Pre) and after (Post) running the ComBat function in ChAMP pipeline removes the batch effects such as “Slide” variations as shown here. **Supplementary Figure 3.** Global distribution of the differentially methylated CGs that showed correlation with Gleason score in combined cohort. 1181 significant (*p <0.05*) differentially methylated CGs that had 10% methylation difference (beta value) in T cell DNA between PCa patients (with Gleason score 9) and in healthy controls were taken and input into IGV browser. Each row represents a Gleason score in a sequence of 6, 7, 8 and 9 while the first row is Chromosome numbers (1-22, X and Y). Hypomethylation (Blue) and hypermethylation (Red). **Supplementary Figure 4.** Venn diagram showing the overlap of CGs between breast, liver and prostate cancer studies. Methylated CGs in T cell DNA that were correlating with Gleason score in this study were overlapped with CGs that were correlating with breast cancer and liver cancer progression in breast and liver cancer studies, respectively. ns, not significant; ****, *p <0.0001*. **Supplementary Figure 5.** Global distribution of the differentially methylated regions (DMRs). DMRs of the T cell DNA between the PCa and healthy control obtained using DMR function in the ChAMP pipeline. The data for the IGV input was obtained from Supplementary Table 3. The first row represents Chromosome numbers (1-22, X and Y), second row shows DMR positions and the last row shows the reference genome H19. **Supplementary Figure 6.** Correlation analysis of percentage tumor volume and Gleason score. The percentage tumor volume was predicted from core prostate biopsies as described in Methods. Negative-biopsy healthy controls had no tumor and therefore assigned a 0% tumor volume and a Gleason score of 0. (A) Linear regression analysis of the percentage tumor volume of the PCa patients with the Gleason score. The red dotted lines show the 95% confidence band of the line of best fit. (B) Non-linear regression analysis of the percentage tumor volume of the PCa patients with Gleason score. The equation for the graph was an exponential growth curve with least square method as the fitting method. **Supplementary Figure 7.** Identification and validation of cg02701909 as a DNA methylation marker using Illumina EPIC (850K) and pyrosequencing. (A-B) Percentage methylation of T cell DNA at cg02701909 plotted as; (A) Ctrl (Control, Blue) and PCa (PCa, Red); (B) Ctrl (Control, Blue), LGPCa (Low Gleason PCa, Red) and HGPCa (High Gleason PCa, Green). The number of samples for pyrosequencing were; Ctrl (n = 9), LGPCa (n = 9) and HGPCa (n = 9). Statistical significance was obtained in GraphPad Prism and are represented by asterisks (*P < 0.05; **P < 0.01, ***P < 0.001, ****P < 0.0001). (C) Correlation graph between Illumina EPIC (850K) and pyrosequencing.

**Additional file 2: Supplementary Table 1.** List of negative-biopsy healthy controls and positive-biopsy PCa patient sample ids with their respective Gleason score.

**Additional file 3: Supplementary Table 2.** List of the primers used for pyrosequencing and amplicon sequencing (Illumina MiSeq system).

**Additional file 4: Supplementary Table 3.** Differentially methylated regions (DMRs) between the PCa T cell DNA and healthy control T cell DNA. The data was obtained using DMR function in the ChAMP pipeline and input into the IGV browser to identify nearby genes and location of each DMR manually. Reference genome H19 was used for alignment. TSS, Transcription start site.

**Additional file 5.**

**Additional file 6.**

**Additional file 7.**

## Data Availability

All data generated or analysed during this study are included in this published article [and its supplementary information files].

## Abbreviations

PCa: Prostate Cancer
LGPCa: Low Gleason Prostate Cancer
HGPCa: High Gleason Prostate Cancer
ChAMP: Chip Analysis Methylation Pipeline
DMP: Differentially methylated probes
BH: Benjamini-Hochberg
HCC: Hepatocellular carcinoma
PCR: Polymerase Chain Reaction
BMIQ: Beta-Mixture Quantile
SVD: Singular Value Decomposition
FDR: False Discovery Rate
IGV: Integrative Genomics Viewer
ROC: Receiver Operating Characteristic
AUC: Area Under Curve
FANTOM5: Functional Annotation of the Mammalian Genome 5
ENCODE: The Encyclopedia of DNA Elements
PMS: Polygenic Methylation Score
