# Supplementary material for: DNA methylation signatures of Prostate Cancer in peripheral T-cells
Source: BMC Cancer. 2020 Jun 23;20:588. doi: 10.1186/s12885-020-07078-8 (PMC7310561; doi:10.1186/s12885-020-07078-8)

Supplementary Figure 1

A

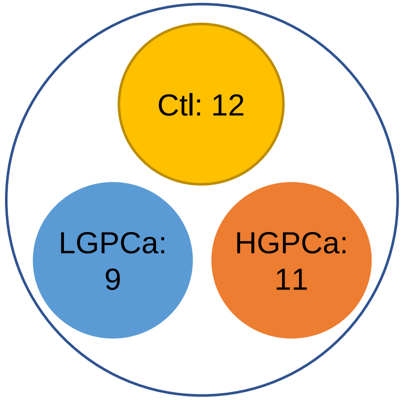

B

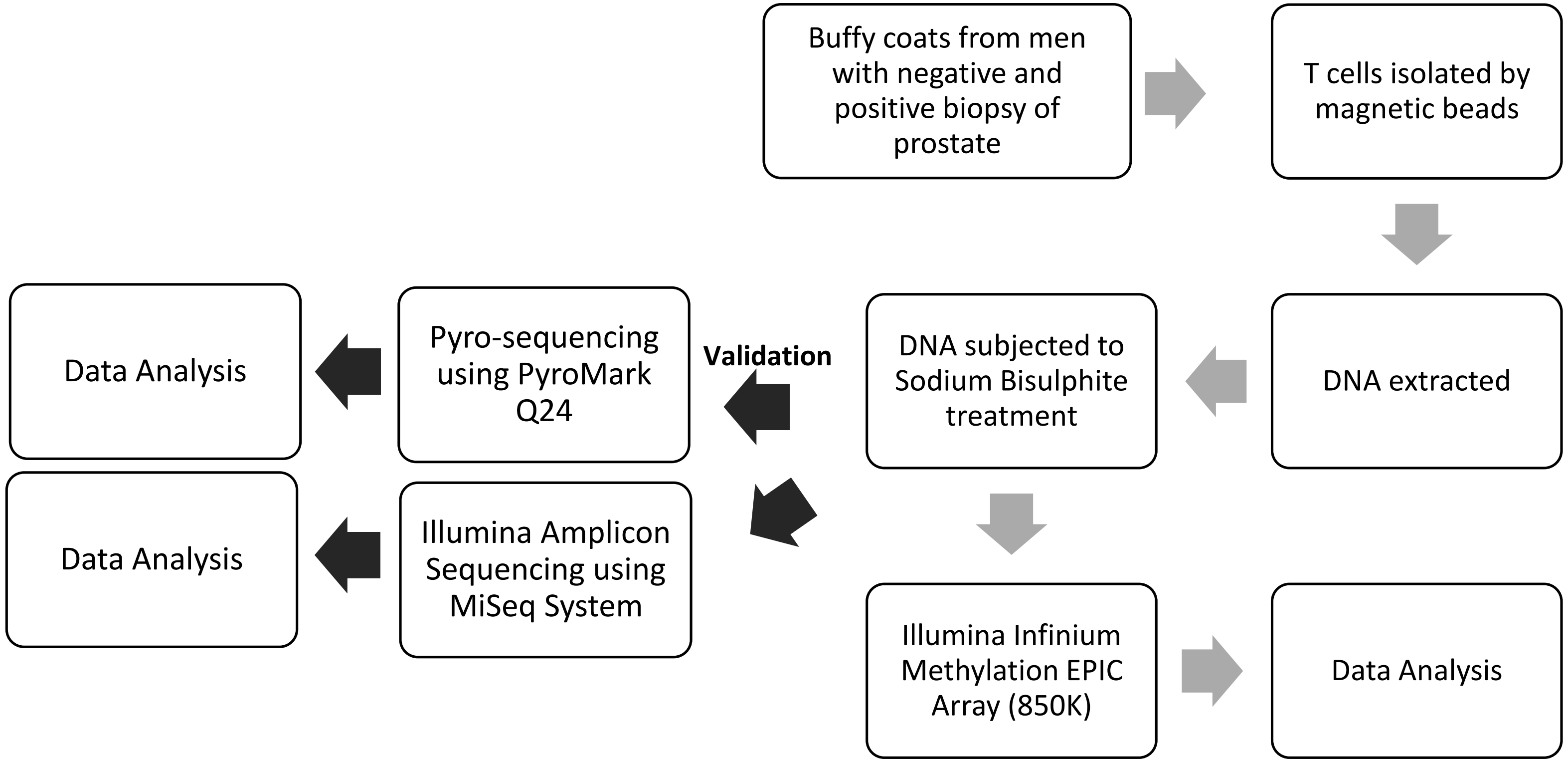

Supplementary Figure 2

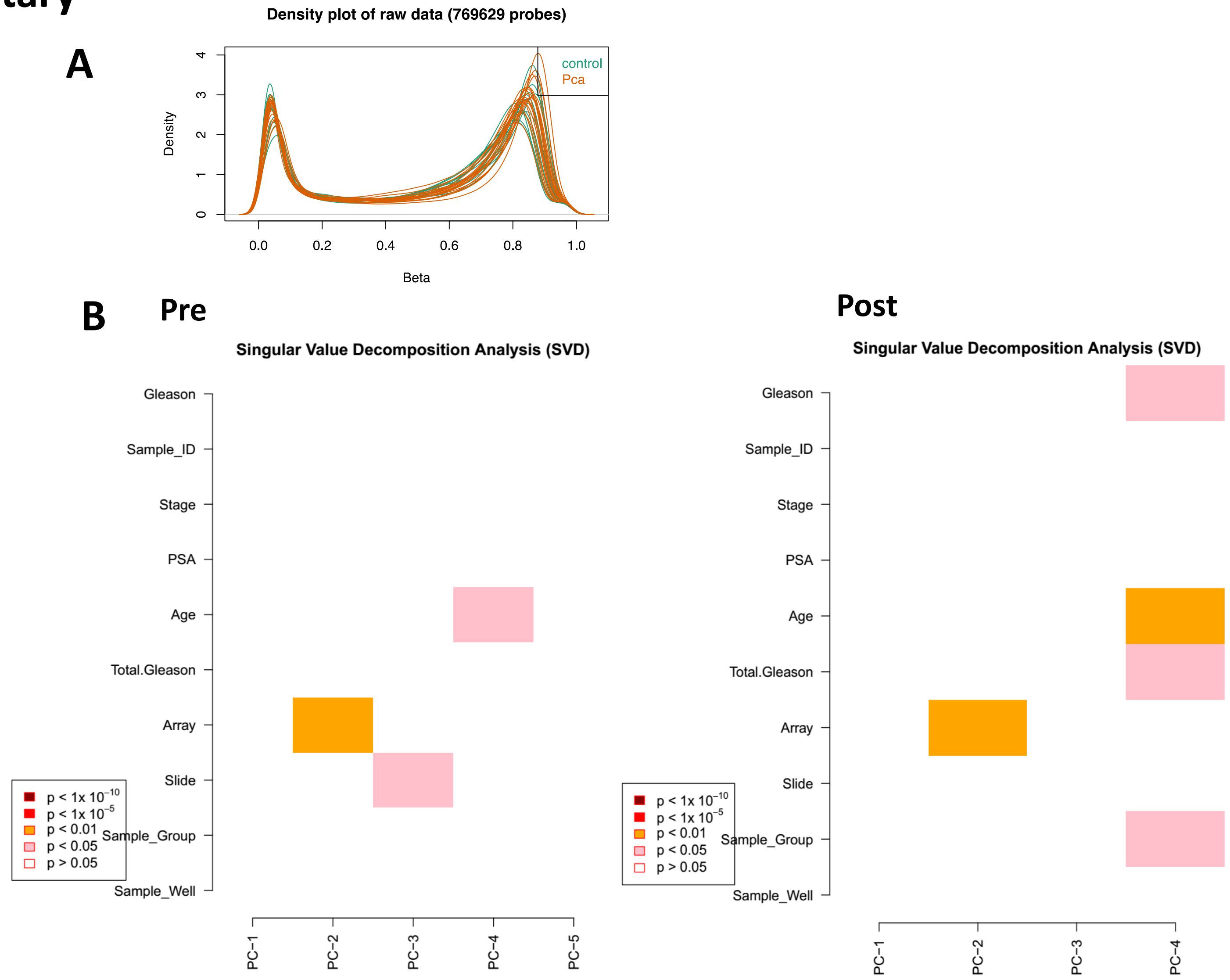

Supplementary Figure 3

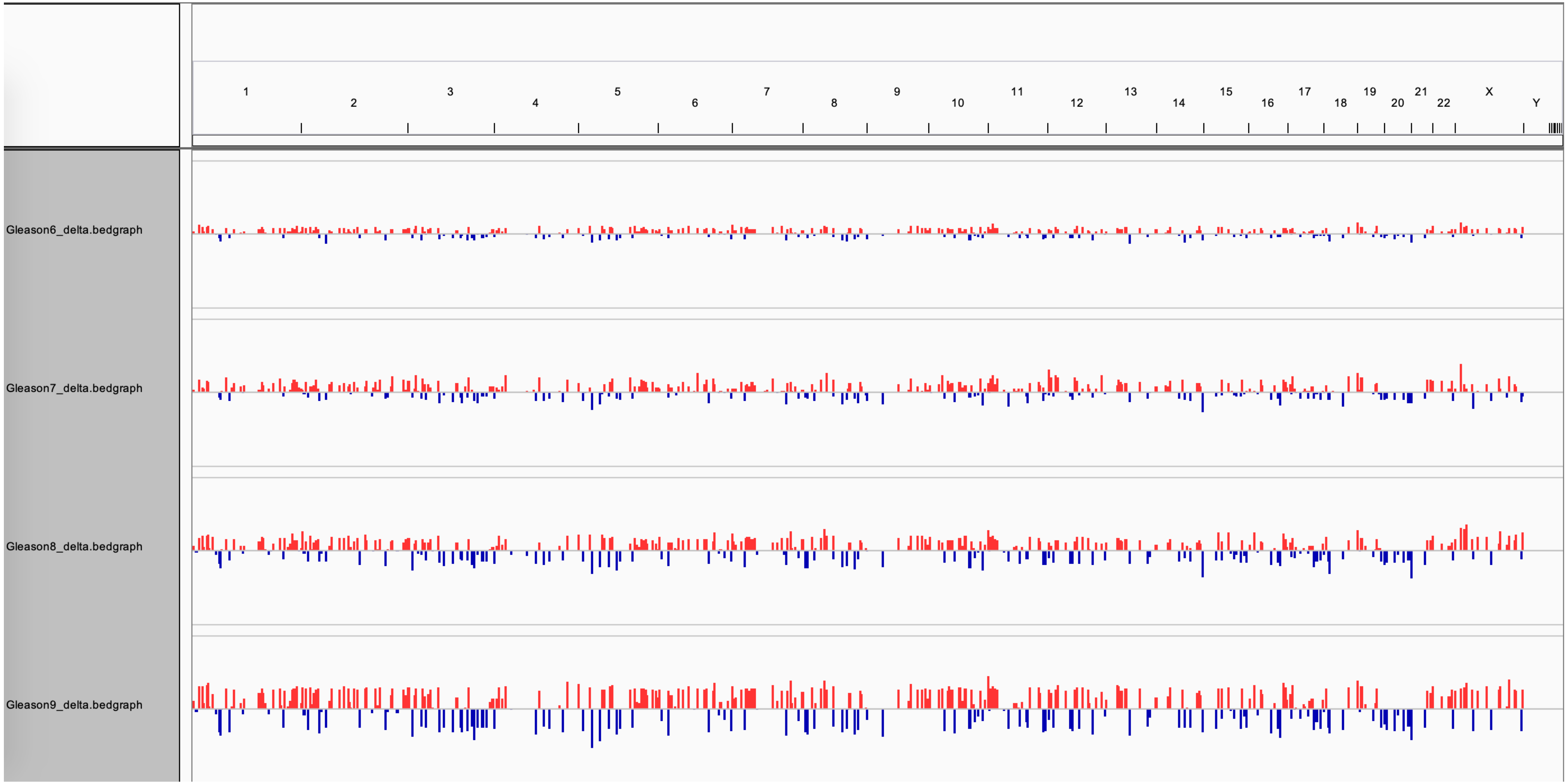

Supplementary Figure 4

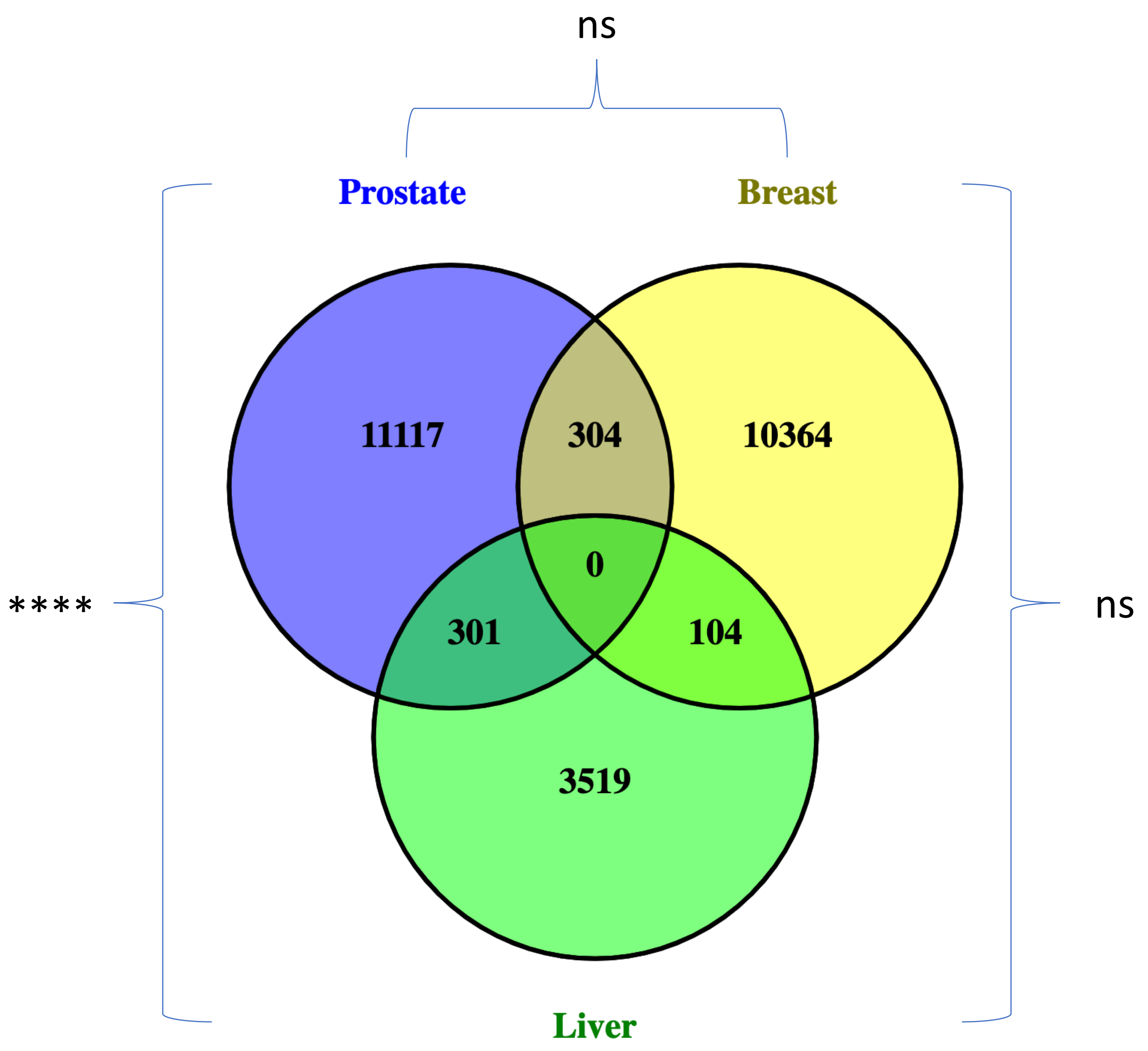

Supplementary Figure 5

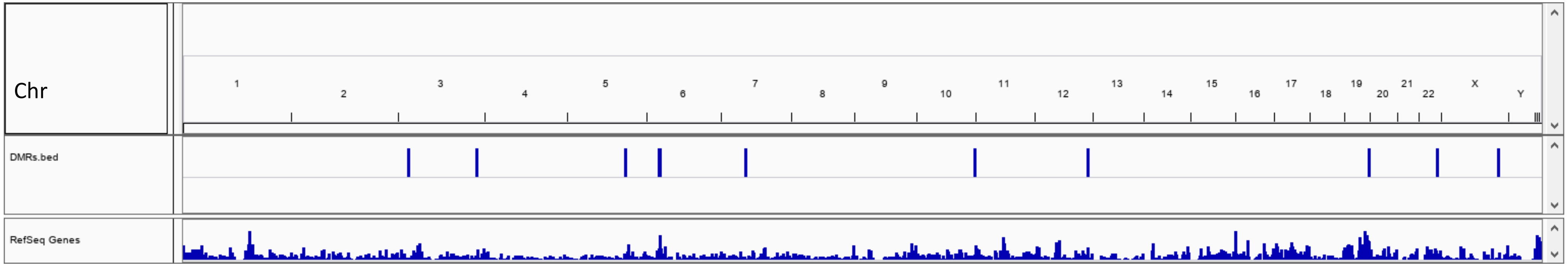

Supplementary Figure 6

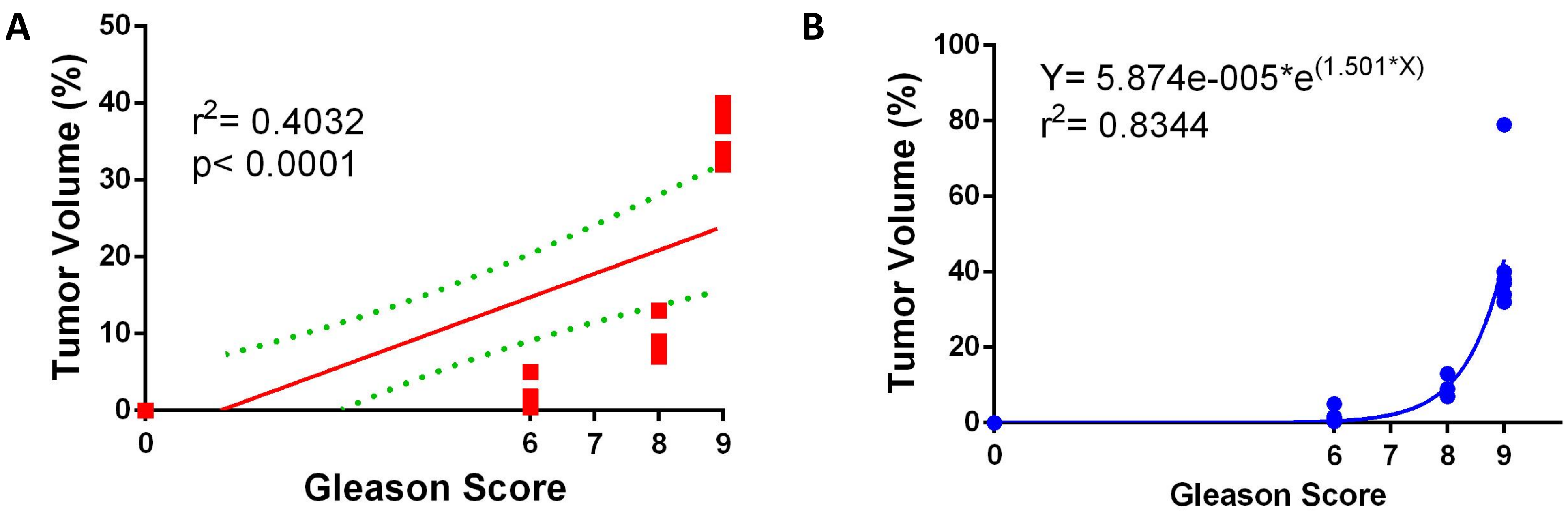

Supplementary Figure 7

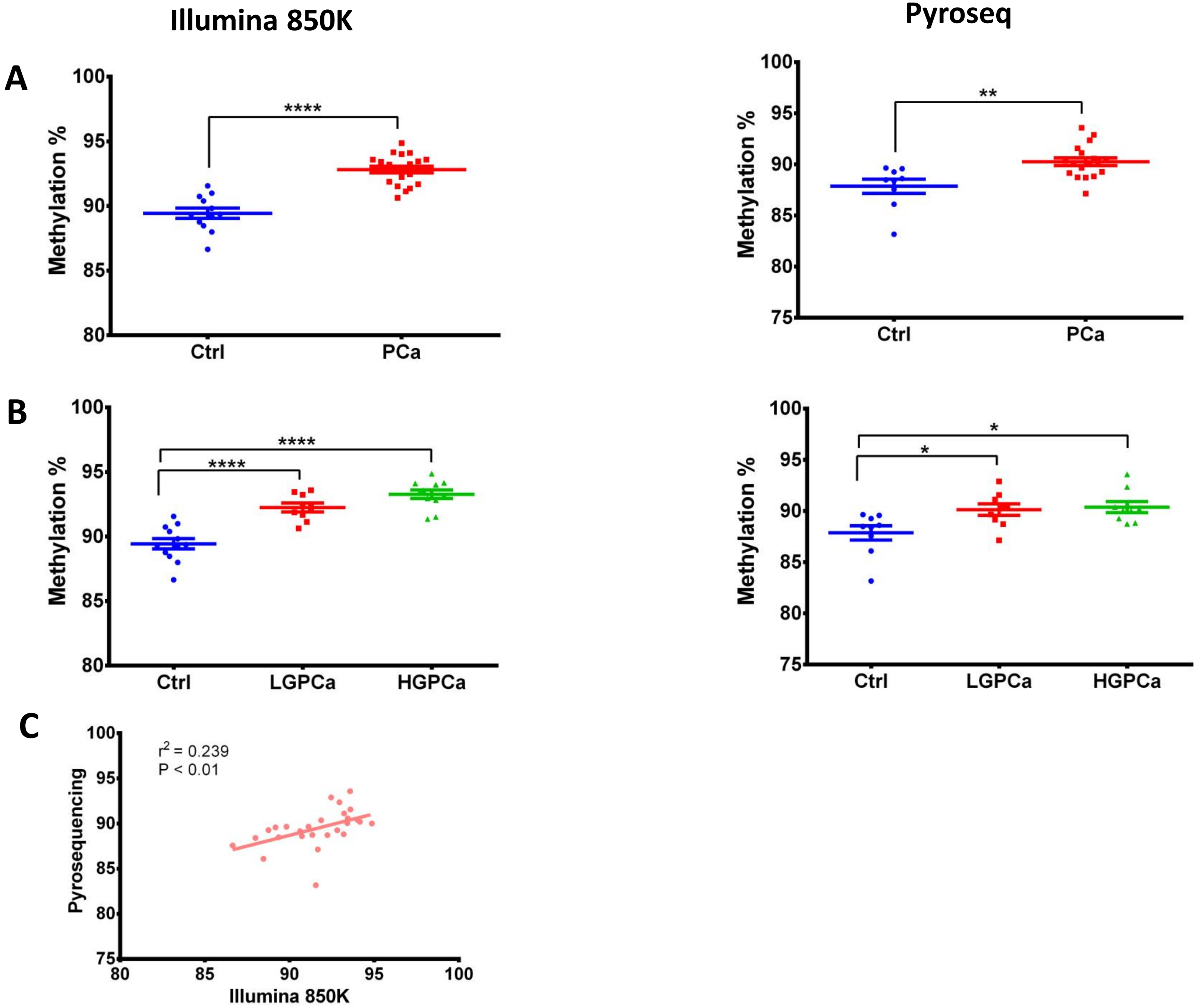

Supplement: Supplementary file 1 — Additional file 1: Supplementary Figure 1. (A) The number of T cell DNA samples used in the cohort 1. Ctrl, Negative-biopsy healthy control; LGPCa, Positive-biopsy low Gleason PCa; HGPCa, Positive biopsy high Gleason PCa. (B) Outline of DNA methylation signature determination in DNA of peripheral T cells in buffy coat of PCa patients and healthy controls using Illumina Infinium EPIC methylation array followed by validation with Illumina MiSeq and Pyrosequencing platforms. Supplementary Figure 2. Quality control (QC) of the data from Illumina Infinium EPIC methylation array using QC in ChAMP pipeline. (A) Density plot of raw data showing the beta distributions for each healthy control and PCa sample. None of the samples deviate from each other. (B) Singular value decomposition analysis (SVD) plot shows the significant components of variation in T cell DNA methylation. SVD analysis before (Pre) and after (Post) running the ComBat function in ChAMP pipeline removes the batch effects such as “Slide” variations as shown here. Supplementary Figure 3. Global distribution of the differentially methylated CGs that showed correlation with Gleason score in combined cohort. 1181 significant (p <0.05) differentially methylated CGs that had 10% methylation difference (beta value) in T cell DNA between PCa patients (with Gleason score 9) and in healthy controls were taken and input into IGV browser. Each row represents a Gleason score in a sequence of 6, 7, 8 and 9 while the first row is Chromosome numbers (1-22, X and Y). Hypomethylation (Blue) and hypermethylation (Red). Supplementary Figure 4. Venn diagram showing the overlap of CGs between breast, liver and prostate cancer studies. Methylated CGs in T cell DNA that were correlating with Gleason score in this study were overlapped with CGs that were correlating with breast cancer and liver cancer progression in breast and liver cancer studies, respectively. ns, not significant; ****, p <0.0001. Supplementary Figure 5. Global di [file 12885_2020_7078_MOESM1_ESM.pdf]
